# Editorial: Computational Solutions for Microbiome and Metagenomics Sequencing Analyses

**DOI:** 10.3389/fmolb.2021.698384

**Published:** 2021-07-30

**Authors:** Md. Selim Reza, YunPeng Cai, Lu Zhang, Xingyu Zhang, Yanjie Wei

**Affiliations:** ^1^ Centre for High Performance Computing, Joint Engineering Research Center for Health Big Data Intelligent Analysis Technology, Shenzhen Institutes of Advanced Technology, Chinese Academy of Sciences, Shenzhen, China; ^2^ Shenzhen Institutes of Advanced Technology, Chinese Academy of Sciences (CAS), Shenzhen, China; ^3^ Department of Computer Science, Hong Kong Baptist University, Kowloon, Hong Kong, SAR China; ^4^ University of Pittsburgh Medical Center, Pittsburgh, PA, United States

**Keywords:** microbiome, metagenomics, taxonomic classification, 16S rRNA sequencing, machine learning

Microorganisms present a huge potential genomic deposit for biosciences, with the human microbiota alone possessing 300 times the number of human genes. This includes genes related to biological functions such as high-efficient enzymes, signal metabolites, and nutrition that are important to human health.

Microbes play key roles in the biology of all living organisms (humans, animals, plants) and they can provide clues to develop new therapies and remediation strategies. Collecting microbiome sequence data has become simpler and less expensive, resulting in an unprecedented increase in the volume of data available for research. With this outburst has come an urgent need for advanced computing resources to help make sense of these massive datasets. The sophistication of microbiome-host-environment-interactions and the vast scale of datasets make it an interesting and important research area. The goal of this special issue “Computational Solutions for Microbiome and Metagenomics Sequencing Analyses” is to address the computational challenges faced by large-scale microbiome profiling data processing in single or multiple aspects such as characterization of microbial community, taxonomic binning, computational methods and key biomarker discovery. Overall this issue accepted 5 papers which report the latest progress on sequence alignment for RNA-seq data, 16S rRNA analysis for disease prediction and cancers antibiotic resistance analysis by metagenomics approach.

Sequence alignment is one of the most important steps in the processing of next-generation sequencing data, and the precision of the alignment has a big effect on downstream applications including variant calling, eQTL analysis, RNA-seq abundance estimation, and metagenomic analysis. Quan et al. developed an MAM (maximal approximate match) algorithm, a novel sequence alignment algorithm that can better search repetitive datasets as opposed to the MEM (maximal exact match) based methods. The efficient alignment is realized via using a modified BWT (Burrows-Wheeler transform) structure for MAM-index construction, MAMs for reducing the candidate locations and affine-gap-penalty dynamic programming to extend all seeds. MAM performs much faster with similar accuracy and sensitivity, at a cost of higher memory demand.

Two articles included in this research topic consider amplicon analysis, a computational technique for taxonomic profiles of the fecal microbiome samples based on 16S rRNA sequencing data. Using metabolic phenotype information (such as metabolism of sugars, amino acids and vitamins), Iablokov et al. developed a novel computational pipeline that can distinguish the microbiome profiles between IBD patients and healthy controls from 16S rRNA sequencing data. The feature engineering technique with the microbial phenotype-based classification methods allows authors to identify new microbiome signatures and provide interpretable insights into the host–microbiome mechanisms of disease that would not be possible with a taxonomy-based approach. Song et al. described a comprehensive comparison of commonly used machine learning models for taxonomic annotation using 16S rRNA sequence data from the V4 hypervariable region. In this study, the authors examined more than 1,000 different variations of three clustering/counting [Operational Taxonomic Unit (OTU), Amplicon Sequence Variants (ASV), and k-mers] techniques, as well as multiple options for normalization and filtering, taxonomic classification, and the use of more than ten different ML approaches for phenotype prediction. According to the author, the method based on k-mers can distinguish between disease and control, as well as provide comparatively high-quality predictions with OTU/ASV and genus assignment approaches. They also discussed the benefits and drawbacks of combinations in analysis methods and provided general insights that can be used as a reference for future trait-prediction studies using microbiome data.

A comprehensive microbiome analysis of blood and cancer-related tissues in cancer patients/controls, revealed the microbiome’s characteristic distribution across various cancer subtypes and their possible contributions to tumourigenesis processes (Poore et al., 2020). Chen et al. reanalyzed the microbiome data of blood and cancer-associated tissues in cancer patients/controls by Poore et al. (2020). Using advanced feature selection techniques with machine learning methods, Chen et al. confirmed that unique microbial signatures contribute to the classification of multiple tumor subtypes, while Poore and his colleagues discovered that these microbiome signatures can be used for discriminating one cancer type versus all others and tumour versus normal cancer. Chen et al. further analyzed/identified the interpretable classification rules for cancer microbiome discrimination by relying on the systematic analysis of microbiome profiling data. For example, Chen et al. established specific rules for ovarian cancers and the high abundances of Oribacterium and Selenomonas are predicted to be correlated with the initiation and the progression of ovarian cancers. Qiu et al. analyzed the antibiotic resistance gene (ARG) profiles of 2,037 samples from 12 countries, focusing on geographic and disease effects on the human gut resistome. Researchers used non-metric multidimensional scaling analysis based on the abundance matrix of the subtypes to visualize ARG structure, then conducted a permutational multivariate analysis of variance. The result confirmed that geographic origin was a strong influential factor impacting on ARGs and suggested a significant difference across health states with a *p*-value of 0.001, although it was much weaker than geographic origin.

To explore this issue, the first paper developed a novel sequence alignment algorithm (MAM) that can better search repetitive datasets as opposed to the MEM based methods. Other papers proposed advanced algorithms to study the interactions/associations between human gut microbiome and the homeostasis of the disease, carcinogenesis, or resistome. Biologically relevant features are important for these algorithms to improve the prediction performance, which is demonstrated by Iablokov et al. for using metabolic phenotype information in their microbial phenotype-based classification method. In addition, classifying possible interactions among the microbiome and carcinogenesis allows researchers to investigate the complex microenvironment mechanisms associated to carcinogenesis. In these papers, many useful insights/implications have been concluded, for example: the importance of ARG is identified for disease research by a comprehensive study of human gut resistome. All of the findings demonstrate the use of an expert scheme, with the main goal of identifying new biomarkers and their interpretable classification rules for microbiome research. We believe that this issue will provide a valuable resource for researchers interested in the fascinating and quickly expanding field of microbiome study.
